# Editorial: Molecular informatics in precision medicine

**DOI:** 10.3389/fmed.2024.1526520

**Published:** 2024-12-03

**Authors:** Sayed AbdulAzeez, Hari S. Sharma, J. Francis Borgio

**Affiliations:** ^1^Department of Genetic Research, Institute for Research and Medical Consultations (IRMC), Imam Abdulrahman Bin Faisal University, Dammam, Saudi Arabia; ^2^Department of Pathology and Clinical Bioinformatics, Erasmus MC, University Medical Center, Rotterdam, Netherlands

**Keywords:** bioinformatics, personalized medicine, next-generation sequence (NGS), sarcoidosis, biomarker, recurrent pregnancy loss

## 1 Introduction

The emerging concept in medicine shifts toward precision medicine personalized to an individual's unique genetic makeup and environmental factors. By integrating advanced molecular technologies such as genomics, transcriptomics, proteomics, metabolomics and microbiomics we can unlock the potential to revolutionize healthcare. Molecular informatics plays a crucial role in this transformation. By analyzing vast amounts of biological data, researchers can identify genetic markers, predict disease risk and develop personalized treatment strategies. This Research Topic deals the latest advancements in molecular informatics, exploring how these technologies can be connected to improve patient outcomes. Key areas of focus include (i) next-generation sequencing, a leveraging cutting-edge sequencing technologies to unravel complex genetic variations, (ii) computer-aided drug discovery for utilizing computational tools to accelerate drug discovery and development, (iii) molecular modeling and simulation for simulating biological processes at the molecular level to gain insights into disease mechanisms and (iv) bioinformatics specially applying computational methods to analyze and interpret biological data. Through a comprehensive exploration of these topics, this collection of articles aims to provide a valuable resource for researchers, clinicians and industry professionals working at the forefront of precision medicine. By understanding the power of molecular informatics, we can move closer to a future where healthcare is truly personalized.

## 2 Contributions

A total of nine articles including one review and eight original research articles have been published on this Research Topic. Hu et al. revealed the shared pathogenic mechanisms between type 2 diabetes mellitus and ulcerative colitis using comprehensive strategy merging bioinformatics and machine learning. Authors sourced data from the Gene Expression Omnibus database and targets of Gegen Qinlian decoction were identified using PharmMapper and SwissTargetPrediction. Targets associated with type 2 diabetes mellitus and ulcerative colitis were compiled from various databases. Weighted gene co-expression network analysis, single-cell sequencing analysis, immune infiltration analysis, machine learning, DEG analysis and network pharmacology were the six analysis included in the study. Results showed that the co-morbidity between type 2 diabetes mellitus and ulcerative colitis is primarily associated with immune-inflammatory pathways, including IL-17, TNF, chemokine and toll-like receptor signaling pathways. Machine learning studies identified IGFBP3 as a biomarker for Gegen Qinlian Decoction in treating type 2 diabetes mellitus, while BACE2, EPHB4 and EPHA2 emerged as biomarkers for Gegen Qinlian decoction in ulcerative colitis treatment. The study provides insights into the shared pathogenesis of type 2 diabetes mellitus and ulcerative colitis and proposes novel targets and therapeutic strategies.

Ye et al. reported the presence of mutations in the *ANXA4* gene in patients with recurrent spontaneous abortion (RSA). Variants were annotated and filtered and the pathogenicity of mutations was predicted using various tools. Through whole exome sequencing an *ANXA4* mutation (p.G8D) was identified in one of the 325 samples from recurrent spontaneous abortion patients. This amino acid change was highly conserved among vertebrate species and predicted to be deleterious. Cell adhesion, migration and invasion were all shown to be inhibited by this mutation in functional experiments. The recently discovered *ANXA4* mutation might have important implications for genetic testing and the pathophysiology of recurrent spontaneous abortions. Another interesting bioinformatic meta-analysis by van Wijck et al. exposed a concise gene signature in pathogenesis of sarcoidosis. Expression datasets of sarcoidosis have uncovered both new and previously well-known genes that may be involved in type I and type II interferon-mediated signaling pathways. Cytokines like interferons and STAT1 were upregulated, but eukaryotic initiation factor 2 signaling was downregulated in expression datasets of sarcoidosis according to *in silico* functional analysis. A key role in pathogenesis of sarcoidosis may be attributed to the unique upregulation of matrix metallopeptidase 12 in afflicted tissues. The authors provided additional evidence on the emerging reported evidence to use JAK inhibitors as a targeted treatment strategy in patients with sarcoidosis. Tissue-specific signatures of genes like *MMP12, CXCR6* and *SNTB2* observed by the authors suggested that these genes might involve in granuloma formation and progression. It is imperative to do more transcriptome investigations in order to validate the observation from bioinformatic meta-analysis by van Wijck et al..

Shi et al. analyzed the adverse event reports related to immune checkpoint inhibitors such as protein-1 (PD-1) and its ligand (PD-L1). They collected 5,322 reports from the United States food and drug administration adverse event reporting system regarding the adverse event reports related to protein-1 and its ligand inhibitors. The study observed that except for pembrolizumab, five PD-1/PD-L1 inhibitors were associated with serious side effects on the endocrine glands. The majority of patients experienced adverse events between 30 and 365 days with a median time of 61 days. The majority of patients experienced prolonged hospitalization in over 40% and death in over 10% of cases after administration of nivolumab, pembrolizumab, or durvalumab. The authors concluded that men aged ≥65 years should be concerned about endocrine-related adverse events and emphasize the importance of addressing these issues when using these PD-1/PD-L1 inhibitors. Another study by Ni et al. uses a network pharmacology approach to predict the active ingredients of *Ixeris chinensis*, targets of action, and possible interventions in diseases. The authors utilized various databases and software to predict active ingredients, target genes, protein interactions and signaling pathways. The results revealed 12 effective components of *I. chinensis* and 40 key targets, including AKT1, EGFR, TNF, SRC and ESR1. Molecular docking analysis revealed that the main active components of *I. chinensis* can bind well with key targets. The study also provides a basis for research on *I. chinensis* treatment pathways for related diseases and subsequent drug development. The study further demonstrated the feasibility of *I. chinensis* as a therapeutic agent for many diseases and established a foundation for investigating the specific mechanisms of treating diseases and the development of novel pharmaceuticals. However, more *in vitro* and *in vivo* experiments are needed to verify these observations.

Behairy et al. investigated the disease susceptibility of the mannose-binding lectin (*MBL*) mutation (rs1800450) in the development of vitiligo and psoriasis. This observational study examined the *MBL2* gene at codon 54 using real-time PCR and computational modeling of the single nucleotide polymorphism. All genetic association models found no evidence that rs1800450 significantly impact the risk of psoriasis or vitiligo disease. The study also found no significant correlation with rs1800450 on the clinicopathological features of both psoriasis and vitiligo. The rs1800450 SNP on the *MBL2* gene was not associated with autoimmune skin disorders risk in Egyptian adults. The data further supports that *MBL2* is redundant and does not significantly affect autoimmune skin disorders. Hemagglutinin is an important element in influenza virus infection, making it a potential target for therapeutic and vaccine development. Zou et al. aimed to create a computational model for identifying hemagglutinin using a benchmark dataset of 106 hemagglutinin and 106 non-hemagglutinin sequences from UniProt. Using the stacking approach, Zou et al. created an integrated classifier model with an accuracy of 95.85% in 5-fold cross-validation and 93.18% in the independent test. The high prediction accuracy makes it useful for biochemical researchers studying hemagglutinin.

Ping et al. developed an amplification analysis using double allele-specific binding primers for accurate measurement of antihypertensive pharmacogenomics. The researchers used quadruplex quantitative PCR (qPCR) and triplex qPCR analysis for genotyping. Mismatch allele-specific F-primers were validated through agreement analysis/reproducibility evaluation. Seven pairs of primers were successfully selected, with amplification efficiency ranging (except for ADRB1) from 0.9 to 1.1 with the coefficient of variation (CV) was < 5%. The study concluded that multiplex amplification analysis using screened allele-specific binding primers is a simple, reliable and accurate tool for guiding drug delivery in antihypertensive personalized treatment. Wen et al. compared the effectiveness of metagenomic next-generation sequencing and conventional microbiological tests in diagnosing pulmonary infections in patients with systemic autoimmune rheumatic diseases receiving immunosuppressant therapy. The study involved reviewing the medical records of 40 patients with pulmonary infections and systemic autoimmune rheumatic diseases treated with immunosuppressants or corticosteroids. Bronchoalveolar lavage fluid samples were collected and examined by metagenomic next-generation sequencing and conventional microbiological tests. The results showed that metagenomic next-generation sequencing had a higher diagnostic accuracy for detecting co-infections with bacteria and fungi and single infections with fungi. The detection rate of co-infection was significantly higher for metagenomic next-generation sequencing than conventional microbiological tests. The study concluded that metagenomic next-generation sequencing's superior accuracy can help ensure timely adjustment of treatment regimens, improving diagnosis and outcomes in patients with systemic autoimmune rheumatic diseases treated with immunosuppressants.

## 3 Conclusion

The Research Topic “*Molecular informatics in precision medicine*” presents a compelling collection of research articles that highlight the transformative power of bioinformatics and computational biology in advancing personalized healthcare. One of the key themes explored in this Research Topic is the identification of novel biomarkers and therapeutic targets for complex diseases. For instance, the study by Hu et al. revealed shared pathogenic mechanisms between type 2 diabetes mellitus and ulcerative colitis, suggesting potential therapeutic strategies. Similarly, Ye et al. identified a novel mutation in the *ANXA4* gene associated with recurrent spontaneous abortion, opening new avenues for genetic testing and targeted therapies. Another important theme is the application of advanced computational methods to analyze large-scale genomic and clinical data. The study by van Wijck et al. utilized bioinformatic meta-analysis to identify key genes involved in the pathogenesis of sarcoidosis, providing insights into potential therapeutic interventions. The increasing use of immune checkpoint inhibitors has led to significant advancements in cancer therapy. However drugs used are associated with adverse effects. Shi et al. investigated the adverse event reports related to PD-1 and PD-L1 inhibitors, highlighting the importance of careful monitoring and management of treatment-related side effects. In addition to human diseases, the Research Topic also explores into the application of computational methods in drug discovery and development. Ni et al. used network pharmacology to identify potential therapeutic targets for *Ixeris chinensis*, a traditional Chinese medicine. Finally, the Research Topic highlights the potential of emerging technologies like metagenomic next-generation sequencing in improving clinical diagnosis and treatment. Wen et al. demonstrated the superiority of metagenomic sequencing over conventional methods in diagnosing pulmonary infections in immunosuppressed patients.

In conclusion, the articles in this Research Topic showcase the immense potential of molecular informatics in revolutionizing precision medicine. By integrating computational biology with clinical research, we can unlock new insights into disease mechanisms, identify novel biomarkers, and develop personalized treatment strategies. As technology continues to advance, we can expect to see even more groundbreaking discoveries in the field of precision medicine.

